# The PilZ domain of MrkH represents a novel DNA binding motif

**DOI:** 10.1007/s13238-016-0317-y

**Published:** 2016-09-20

**Authors:** Feng Wang, Qing He, Kaixuan Su, Fei Gao, Yan Huang, Zong Lin, Deyu Zhu, Lichuan Gu

**Affiliations:** 1 State Key Laboratory of Microbial Technology, Shandong University, Jinan, 250100 China; 2 Department of Biotechnology and Biomedicine, Yangtze Delta Region Institute of Tsinghua University, Jiaxing, 314006 China

**Dear Editor,**

MrkH is the first characterized c-di-GMP related transcriptional regulator which affects type 3 fimbrial expression in response to cellular c-di-GMP level, and thus plays an important role in the biofilm formation of *Klebsiella pneumoniae* (Murphy and Clegg, 2012; Wilksch et al., 2011). However, how MrkH recognizes c-di-GMP and the target DNA sequence remains obscure.

Here, we determine the crystal structure of MrkH/c-di-GMP complex at 2.3 Å resolution. MrkH adopts a tandem two-domain structure with a canonical YcgR-N/PilZ proteins-fold, it contains two α-helixes (α1, α2) and 18 β-sheets which formed β-barrel 1 and β-barrel 2 (Fig. 1A and 1B). Our size-exclusion chromatography results demonstrate that MrkH forms a stable monomer either in the presence or absence of c-di-GMP (Fig. 1C). A *DALI* search for globally similar proteins revealed that MrkH/c-di-GMP has notable structural homology with PilZ domain proteins (Benach et al., 2007; Holm and Rosenstrom, 2010; Ko et al., 2010). The binding of c-di-GMP to MrkH is very similar to Pp4397 - both MrkH and Pp4397 hold tightly two mutually intercalated c-di-GMP molecules, while VCA0042 combines with only one c-di-GMP (Fig. 1D). Through structure analysis we find that MrkH mainly forms H-bond with two c-di-GMP molecules (Fig. 1D and 1E). The side chains of R107 and R111 also contribute a cation–π interaction with the guanine group of C2E2 and C2E1 respectively. Multiple-sequence alignment revealed these residues are well conserved (Fig. S1). Besides the interactions between protein and ligands, two c-di-GMP molecules are also stabilized by strong base stacking interaction between mutually intercalated guanine groups. We further analyze the interactions between MrkH and c-di-GMP using ITC (Fig. S2A–G). The affinity of MrkH for c-di-GMP is high with *K*_d_ of approximately 0.24 μmol/L using the one site specific binding model(Whitney et al., 2015). MrkH^105-end^ also binds to c-di-GMP efficiently. However, MrkH^116-end^ loses the c-di-GMP binding affinity, which indicates that the connecting loop between two β-barrels is crucial for c-di-GMP binding. R107 forms both H-bond and cation–π interaction with two c-di-GMPs (Fig. 1D), thus R107A mutant has greatly decreased binding affinity for c-di-GMP with a dissociation constant fifteen times higher than that of the wild-type MrkH (3.55 μmol/L vs 0.24 μmol/L). R111A mutant almost completely loses the c-di-GMP binding affinity, indicating that R111 is the most important residue for c-di-GMP binding. MrkH variants bind to c-di-GMP with an approximately stoichiometry of 1:2 (Table S1).

**Figure 1:**
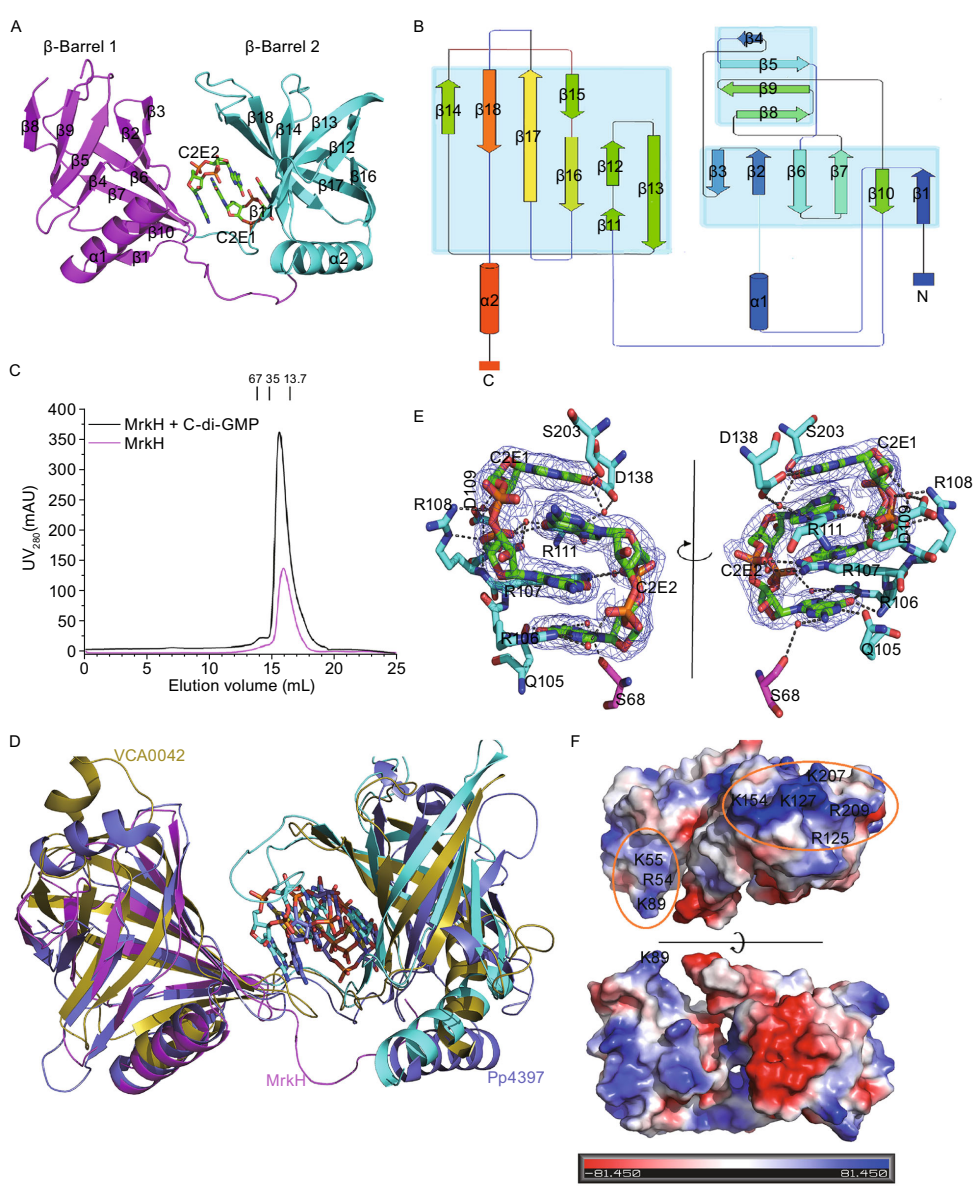
Crystal structure of the MrkH/c-di-GMP complex. (A) Cartoon diagram depicting MrkH, in which YcgR-N domain is shown in magenta and PilZ domain in cyan. Sticks diagram depicting two mutually intercalated c-di-GMP molecules. Secondary-structure elements referred to in the text are labeled. (B) Topology diagram of MrkH was decomposed into domains in accordance with Pro-origami(Stivala et al., 2011), β-Strands are shown as arrows and α-helices as columns. (C) Size-exclusion chromatography of MrkH and MrkH/c-di-GMP complex. The horizontal and vertical axes represent elution volume and ultraviolent absorbance (λ = 280 nm), respectively. (D) Structural comparison of MrkH, PP4397 and VCA0042. The structures of MrkH (YcgR-N is colored magenta and PilZ domain cyan), PP4397 (in sliate) and VCA0042 (in olive) were compared with their YcgR-N domain alignments. The c-di-GMP molecules were shown as sticks (E) The 2*Fo-Fc* electron-density map for two mutually intercalated c-di-GMP molecules is contoured at 1σ. The residues that form H-bonds with c-di-GMP (C2E) are labeled and shown as ball-and-stick models. Water molecules that involved in H-bonds formation are shown as red spheres. Black dotted lines indicate H-bonds. (F) MrkH is shown as surface representation and colored according to their “in vacuum” electrostatics (red for negatively charged regions, and blue for positively charged regions, Pymol). The residues involved in the DNA-binding are labeled and circled by orange ovals

Previous studies have demonstrated that MrkH could bind directly to the promoter of *mrkHI* or *mrkA*, and c-di-GMP molecule promotes the binding of MrkH to the promoter (Tan et al., 2015; Wilksch et al., 2011; Yang et al., 2013), but it is unclear how MrkH recognizes its targets DNA. To address this problem, we incubated MrkH with various DNA fragments for EMSA. We purified recombinant MBP-MrkH, MBP-MrkH-YcgR-N domain (residues 1-104) and MBP-MrkH-PilZ domain (residues 105-end) which were incubated with the unlabeled *mrkHI* and *mrkA* promoter fragments respectively. The evident DNA-protein complex migrations were observed in lanes of MBP-MrkH and MBP-MrkH-PilZ domain (Fig. 2A). It suggests that MrkH binds directly to the *mrkHI* and *mrkA* promoter sequence mainly through its PilZ domain. The EMSA also shows the migration velocity of DNA fragments constantly slow down as protein concentration increases (Fig. 2B) and MrkH-PilZ without MBP tag gives the same result (Fig. 2C). Size-exclusion chromatography analysis of PilZ domain indicates that the oligomeric state of PilZ domain is not affected by protein concentration (Fig. S3A). These observations implied that a long DNA fragment may recruit multiple PilZ domains and resulting nonspecific binding. In order to locate the DNA binding region in MrkH-PilZ domain, the vacuum electrostatics of MrkH/c-di-GMP structure is carefully analyzed. A highly positive charged hump consisting of six basic residues (R125, K127, K154, K163, K207 and R209) is identified (Fig. 1F). Since DNA is a negatively charged, this positively charged region may be critical for DNA binding. To test this hypothesis, we constructed several cognate mutants of MrkH-PilZ domain and performed EMSA with the *mrkA* regulatory fragment. The EMSA result shows that all these mutants lose DNA binding abilities (Fig. S3B). This indicates that these basic residues play a critical role in DNA binding of MrkH-PilZ domain. What’s more, these mutations do not affect the interactions between MrkH and c-di-GMP (Fig. S2H–J). Multiple-sequence alignment shows that these positively charged residues are conserved among many MrkH homologues (Fig. S1), which means that PilZ domain may represent a novel DNA-binding motif.

**Figure 2:**
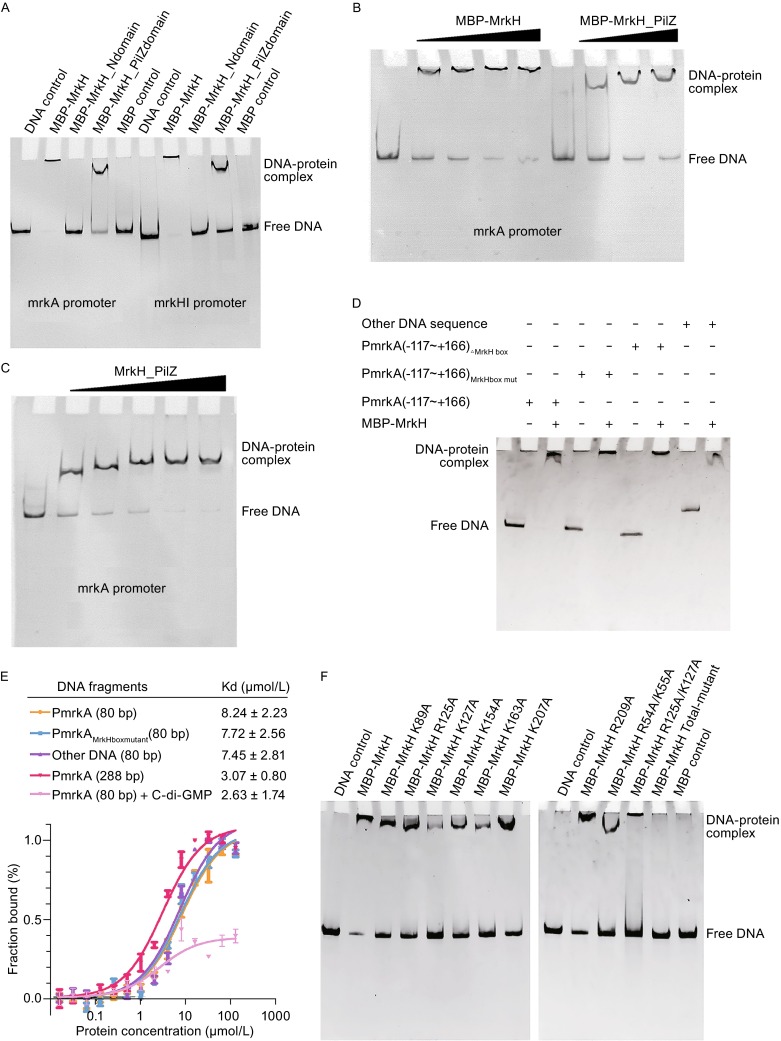

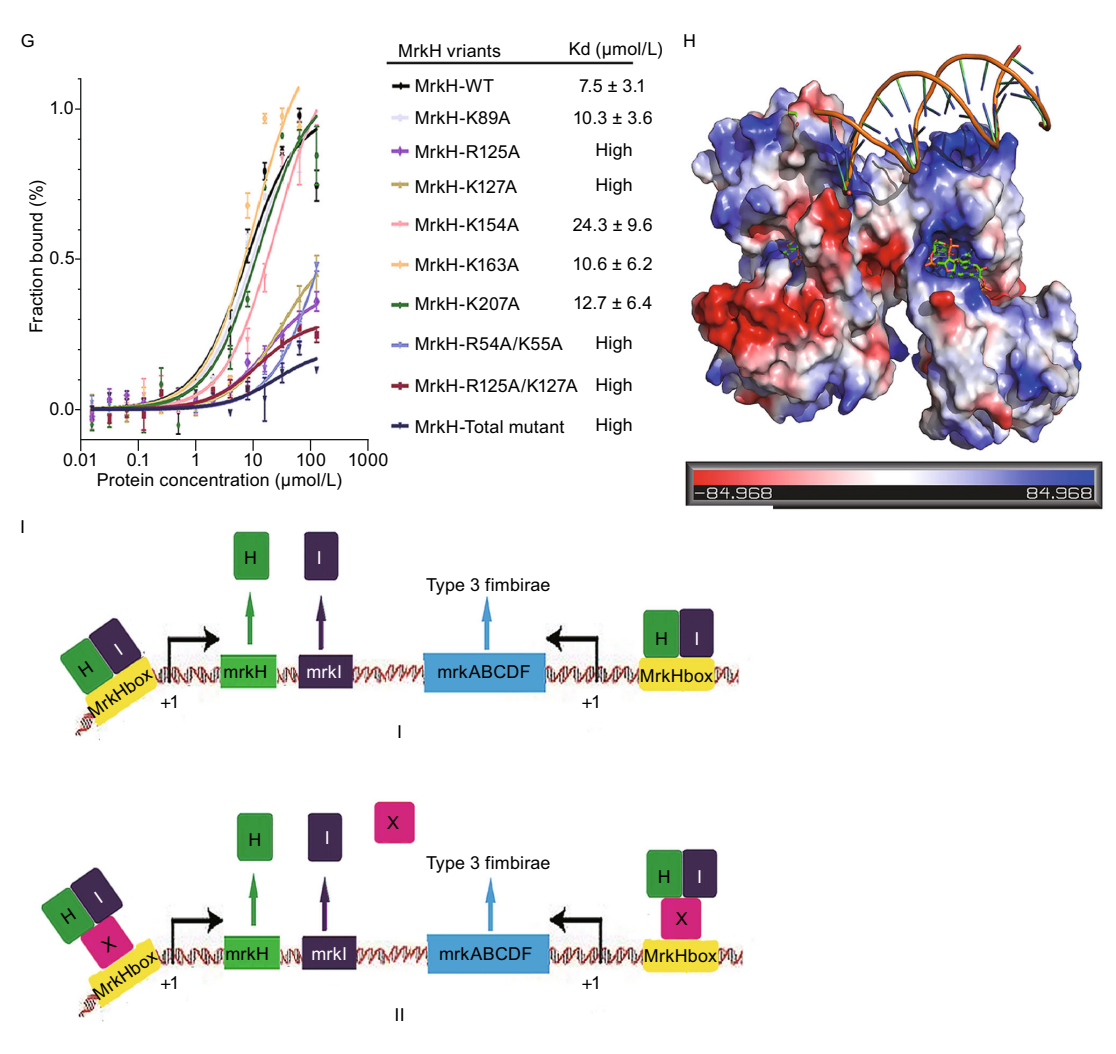
MrkH-PilZ domain is a novel DNA-binding motif. (A) EMSA was performed using a 288 bp *mrkA* promoter containing base pairs −117 to +166 relative to the start site of *mrkA* transcription and 250 bp *mrkHI* promoter containing base pairs −184 to +52 relative to the start site of *mrkH* transcription with MrkH and MrkH two sides domains. (B) EMSA was performed for MBP-MrkH and MBP-MrkH-PilZ with the 288 bp *mrkA* promoter sequence. The same amount of DNA was used for each lane. Free DNA decreases with the increase of protein concentration. (C) EMSA of MrkH-PilZ with the 288-bp *mrkA* promoter sequence. As protein concentration increases, the amount of free DNA drops,the electrophoretic mobility of protein-DNA complex also declines. (D) EMSA was performed for MBP-MrkH with a variety of 288 bp *mrkA* promoter sequences and a random DNA fragment. (E) Fluorescence polarization curves are shown for the binding of MrkH to the FAM-labeled *mrkA* promoter fragments (288 bps from −117 to +166; 80 bps from −117 to −37 and the ‘MrkH box’ mutant; and a random 80 bps DNA). (F) EMSA analysis of MBP-MrkH mutants and the *mrkA* promoter sequence. (G) DNA-binding affinities of wild-type and mutant MrkH. Fluorescence polarization curves are shown for the binding of purified proteins to a FAM-labeled 80 bp *mrkA* promoter fragment (from −117 to −37). (H) The Docking DNA/MrkH complex structure, in the proposed MrkH dimer two separate DNA-binding regions merge into a larger positively charged area binding DNA. (I) The model for MrkHI regulating the expression of type 3 fimbriae in *K. pneumoniae*.: [I]MrkH and MrkI form MrkHI complex which regulates the expression of *mrkABCDF* and *mrkHI*. [II] MrkHI complex recruits a not-yet-indentified protein X forming a ternary complex which regulates the transcription of *mrkABCDF* and *mrkHI*. The corresponding *K*_d_ values are obtained by fitting data to one-site specific binding model, and the error bars represent ± SD for triplicate experiments

It has been reported that MrkH could directly bind to the *mrkA* and *mrkHI* promoter through specifically recognizing the identified palindrome sequence named ‘MrkH box’ in these regions (Tan et al., 2015; Yang et al., 2013). Our data have shown that MrkH binds to dsDNA mainly through its PilZ domain and the longer DNA fragment containing the ‘MrkH box’ could recruit multiple PilZ domains. However, the ‘MrkH box’ is a very short DNA segment which is unlikely to be long enough to recruit multiple MrkH-PilZ domains. To evaluate the role of ‘MrkH box’ for full-length MrkH-DNA interaction, we incubated MBP-MrkH with the *mrkA* regulatory fragment with the ‘MrkH box’ changed or deleted for EMSA. Indeed, MBP-MrkH binds to the *mrkA* regulatory fragment readily, without any specific dependence on ‘MrkH box’. Moreover, it can even bind to a random DNA sequence equally well (Fig. 2D). Fluorescence polarization (FP) was used to further evaluate the interaction of MrkH and different DNA sequences. The FP measurements indicate that MrkH binds the longer *mrkA* regulatory fragment (from −117 to +166) with a *K*_d_ value of 3.07 μmol/L using the one site specific binding model, while MrkH binds to a shorter *mrkA* regulatory fragment (from −117 to −37) with a *K*_d_ value of 8.24 μmol/L. Adding c-di-GMP strengthens the MrkH-DNA interaction to a *K*_d_ value of 2.63 μmol/L (Fig. 2E). Intriguingly, the FP value decreased in the presence of c-di-GMP. This was probably due to the c-di-GMP-induced conformation change of MrkH which resulted in a more compact globular protein molecule with faster rotation. The interaction of MrkH and the 12-bp ‘MrkH box’ sequence proved to be undetectable because FP value change was too little. But we observed that mutations in ‘MrkH box’ did not affect the binding affinity of MrkH to the *mrkA* regulatory fragment. In addition, an 80-bp random DNA sequence binds to MrkH equally well with a *K*_d_ value of 7.45 μmol/L (Fig. 2E). Therefore, it is undoubtedly that MrkH binds to DNA without sequence specificity *in vitro*, and thus binds to longer DNA strand more tightly. The interaction between MrkH and DNA can be significantly strengthened in the presence of c-di-GMP.

We now know that MrkH-PilZ domain has dual functions. It binds c-di-GMP and also represents a novel DNA binding motif. However, MrkH has a higher efficiency in DNA binding than its PilZ domain (Fig. 2A), which suggests the N-terminal domain of MrkH could also contribute to DNA binding. Surface analysis indicates that residues R54, K55 and K89 form a small positive charge cluster on the YcgR-N domain. We engineered these mutation for EMSA analysis. The EMSA results showed that the mutations of MBP-MrkH R54A/K55A and K89A result in weaker DNA-binding, indicating that these residues also play a role in DNA-binding (Fig. 2F). For FP analysis, mutations of K89A, K154A, K163A and K207A had little effect on the DNA-binding affinity of MrkH. However, mutations of R54A/K55A, R125A, K127A and R125A/K127A of MrkH slightly decreased the DNA-binding affinity, and the simultaneous mutation of all these DNA-binding related residues leads to a total loss of DNA-binding affinity (Fig. 2G). CD spectra and SDS-PAGE of these mutants showed that these mutants have maintained stable structures (Fig. S3C and S3D). Given all these data, we think MrkH has two separate DNA-binding regions, with a smaller one on N-terminal domain and a larger one on PilZ domain (Fig. 1F). Crystal packing analysis helps us to identify the MrkH dimer in which makes two separated regions merge into a long region suitable for DNA binding. We used DP-dock (Tuszynska et al., 2015) to predict a MrkH/DNA model and the result shows that the interactions between predicted DNA-binding regions and double-stranded DNA are appropriate (Fig. 2H). We propose that multiple copies of this dimer would tandemly bind to a DNA strand until the whole DNA strand is saturated.

In this study, we obtained the structure of MrkH in complex with c-di-GMP at 2.3 Å resolution and made a starting point for understanding the DNA-binding model of MrkH. Previous studies revealed that MrkH performed its function through specifically recognizing the palindromic ‘MrkH box’ to regulate the transcription of *mrkHI* and *mrkABCDF* cluster (Tan et al., 2015; Yang et al., 2013). Here, we found that MrkH binds to the promoter regulation fragments of *mrkHI* and *mrkA* mainly through its PilZ domain, the YcgR-N domain of MrkH also participates in DNA-binding. Additionally, a long DNA fragment may recruit multiple PilZ domains and MrkH binds to DNA in a sequence-independent manner. *mrkI* is co-transcribed with *mrkH* and encodes a LuxR-type DNA-binding protein, it can regulate type 3 fimbrial production especially in *K. pneumoniae* IApc35 (Johnson et al., 2011). It is possible that MrkH and MrkI form a protein complex to perform the regulatory function. On the basis of previous reports (Christen et al., 2007; Ryan et al., 2012; Tan et al., 2015) and our data, we tentatively propose two models for MrkH regulates the corresponding gene transcriptions. The simple model is that although MrkH binds to DNA un-specifically, once MrkI exists and forms MrkHI complex, this protein complex would bind to ‘MrkH box’ specifically and turn on corresponding gene transcriptions in the presence of c-di-GMP. To test this model, we have made a lot of effort to purify MrkHI complex. Unfortunately the poor behavior of MrkI makes the judgment on this issue impossible. Another model requires a not-yet-identified protein X that can specifically recognize ‘MrkH box’. Once protein X binds to ‘MrkH box’, it will recruit MrkH and MrkI to form an active ternary complex which finally activates transcription of *mrkABCDF* and *mrkHI* in the presence of c-di-GMP (Fig. 2I). So far there is not enough data to tell which model is the correct. In this respect, more *K. pneumoniae* physiology data would help better understand the mechanism of MrkH in detail.

## Electronic supplementary material

Below is the link to the electronic supplementary material.

Supplementary material 1 (PDF 1132 kb)
