# Supplementary material for: The PilZ domain of MrkH represents a novel DNA binding motif
Source: Protein Cell. 2016 Sep 20;7(10):766–72. doi: 10.1007/s13238-016-0317-y (PMC5055493; doi:10.1007/s13238-016-0317-y)
Supplement: Supplementary file 1 — Supplementary material 1 (PDF 1132 kb) [file 13238_2016_317_MOESM1_ESM.pdf]

This supplementary data file contains the following contents: Materials and methods, Supplementary Figures (S1~S3) and Supplementary Tables (S1~S4).

Materials and methods

Gene cloning and mutant construction

The truncated MrkH (1-228) lacking 6 residues from the C-terminus was cloned into the expression vector pET-28a (+) (Novagen) with an N-terminal His_6 tag using the NdeI/XhoI restriction sites and was used for crystallization procedure. MrkH variants, the MrkH-YcgR-N domain (residues 1-104) and the MrkH-PilZ domain (residues 105-end) were cloned into the reconstructed vector pET-28b with an N-terminal MBP fusion tag using the BamhI/XhoI restriction sites. These MBP fusion proteins were used for EMSA. MrkH truncations and variants were also cloned into another reconstructed vector pET-28b with a TEV protease cleavage site at the N-terminus to obtain the untagged proteins using the NdeI/XhoI restriction sites. His_6 tag was removed for protein samples used in ITC and FP measurements. The construction of various mutants were performed using the QuikChange® II site-directed mutagenesis kit (Agilent Technologies) following a published protocol (Xia et al., 2015). The residues selected to be mutated were confirmed by DNA sequencing.

Protein production and purification

All the constructs were transformed into *E. coli* BL21 (DE3) cells for overexpression. Cells were grown in Luria-Bertani medium at 37 ℃ with ampicillin

(Amp; 100 μg•ml^{-1}) and kanamycin (Kan; 50 μg•ml^{-1}), until the cell density reached an $OD_{600\ nm}$ of 0.6–0.8. The overexpression was induced by 0.1 m*M* IPTG (isopropyl-*β*-D-1-thiogalactopyranoside) at 16 ℃ overnight. The bacterial cells were harvested by centrifugation at 6000×g for 15 min and resuspended in 25 m*M* Tris–HCl (pH 8.0), 0.5 *M* NaCl, 10 μg ml^{-1} DNase I and 0.5 m*M* Phenylmethylsulfonyl fluoride (PMSF) for sonication. The supernatant was obtained by centrifugation of the cell lysate at 28370×g for 45 min at 4 ℃ and then applied onto Ni–NTA affinity resin (Qiagen) for His_6 tag which was equilibrated with buffer A (25 m*M* Tris-HCl, pH 8.0, 500 m*M* NaCl). Unbound materials were washed out with one column volume of wash buffer B (25 m*M* Tris-HCl, pH 8.0, 500 m*M* NaCl, 10 m*M* imidazole). The target proteins were eluted with 10 ml buffer C (25 m*M* Tris-HCl pH 8.0, 500 m*M* NaCl, 250 m*M* imidazole). The elutes were then concentrated and loaded onto a Superdex200 size-exclusion column equilibrated with 10 m*M* Tris–HCl pH 8.0, 500 m*M* NaCl. The final concentration of $MrkH^{1-228}$-pET-28a was 6 mg•ml^{-1} and stored at -80 ℃ for crystallization use. For the MBP fusion MrkH and mutants, the salt concentration of all buffers was 100 m*M* instead.

Crystallization, data collection and structure determination

MrkH was incubated with c-di-GMP with a molar ratio of 1:2.5 and crystallization screen was performed with kits from Hampton Research using the sitting-drop vapour diffusion method at 4 ℃. The crystal of MrkH/c-di-GMP complex was observed in a solution containing 0.5 M Ammonium sulfate, 0.1 M Sodium citrate tribasic dihydrate and 1.0 M Lithium sulfate monohydrate after 14 d at 4 ℃. To solve phase problem, the

heavy-atom reagent I3C (Hampton Research) was soaked into the native crystals by addition of 0.5 μl 0.5 M I3C solution directly to the crystallization drop. After 15 min, crystals with pale yellow color were chosen for the next step. All Crystals were flash frozen in liquid nitrogen, with addition of 25% (v/v) ethylene glycol serving as a cryoprotectant.

X-ray diffraction data were collected on beamline BL17U1 at the Shanghai Synchrotron Radiation Facility (SSRF). All data were processed using HKL-2000 (Leslie et al., 2002). The heavy atom soaked crystal was determined by the single-wavelength anomalous dispersion (SAD) phasing. Initial phases were calculated using AutoSol implemented in PHENIX (Adams et al., 2010), and seventeen I3C sites were identified. AutoBuild in PHENIX was used to automatically trace the chain of MrkH, and the molecular replacement was performed to solve the native structure using I3C structure as the search model by running MOLREP in CCP4 (Murshudov et al., 2011). After several rounds of positional refinement alternated with manual model revision using Coot (Emsley et al., 2010) and the refinement programs REFMAC (Murshudov et al., 2011) and Phenix.Refine (Adams et al., 2010), the quality of final model was evaluated using the PROCHECK program (Laskowski et al., 1993). Details of the data-collection and refinement statistics are shown in Table S2. All of the structure figures were rendered by PyMOL (www.pymol.org.)(DeLano, 2002).

Electrophoretic mobility shift assay (EMSA)

DNA-binding activities of different MrkH fragments and mutants were analyzed in electrophoretic mobility shift assay (EMSA). The MBP fusion proteins were used for EMSA. Each unlabeled DNA fragment was incubated with varying amounts of purified MBP-MrkH and MrkH-PilZ domain in 50 mM Tris pH 8.0, 100 mM NaCl, 5 mM MgCl2 in a total volume of 20 μl for 100 min on ice. Proteins were added at least at a 103:1 molar ratio to DNA. Reaction samples were then mixed with the loading buffer and separated in 5% native polyacrylamide gels (37.5:1) in 0.5×TBE buffer for 120 min

at 80 V and 4℃. Protein–DNA complexes were stained with Ethidium bromide and visualized by UV imaging system. The DNA fragments containing regulatory region and mrkHI regulatory region were generated by PCR using synthetic plasmid containing those regulatory sequences as templates. The used primers were listed in Table S3.

Binding studies by isothermal titration calorimetry (ITC)

The dissociation constants (Kd) and thermodynamic parameters of binding reactions between c-di-GMP and MrkH or other mutant proteins without tags were measured by ITC using a ITC200 calorimeter (GE Healthcare). The c-di-GMP (0.5 mM volume in the syringe) was injected into solution of MrkH protein or other mutations (0.15 mM) without His6 tag at 25 °C as 2 μl aliquots at a 120 second interval between injections and was stirred at 1000 rev•min-1. The c-di-GMP and proteins buffer were 10 mM Tris-HCl pH 8.0, 300 mM NaCl, 5 mM MgCl2 and 10％(v/v) Glycerol. ITC data were analyzed by integrating the heat effects after the data were normalized to the amount of injected protein. Equilibrium association constant, binding stoichiometry (N) and enthalpy (ΔH) of the binding reaction were derived by fitting the data to a single binding site model according to previous reports (Ko et al., 2010; Whitney et al., 2015)

Fluorescence Polarization Measurements of DNA-Binding Affinity

Each FAM-labeled DNA (10nM) was mixed with increasing amount of wild-type or a variant of MrkH protein. The mixtures were incubated in buffer containing 10 mM HEPES pH 8.0, 50 mM NaCl for 30 min at 4℃. Fluorescence polarization measurements were performed at 25℃ on a Synergy 4 Microplate Reader (BioTek). Data were fitted with GraphPad Prism 5, and the apparent Kd values were calculated accordingly. The FAM-labeled DNA sequences were listed in Table S4.

Gel-filtration assay

The MrkH and MrkH/c-di-GMP complex were subjected to gel-filtration analysis (Superdex 200 10/300 GL column; GE Healthcare; 10 mM Tris–HCl pH 8.0; 500 mM

NaCl) with or without 50 μM c-di-GMP, the assay was performed at a flow rate of 0.4 ml•min-1 and 0.1 ml of MrkH (about 2.0 mg•ml-1) was injected at 4 ℃. The elution volume of the MrkH/c-di-GMP was 15.71 ml under the conditions of the assay, while the elution volume of MrkH monomer was 15.98 ml. MrkH-PilZ proteins were also analyzed with the above method (except for NaCl concentration of 100 mM).

Supplementary Figures

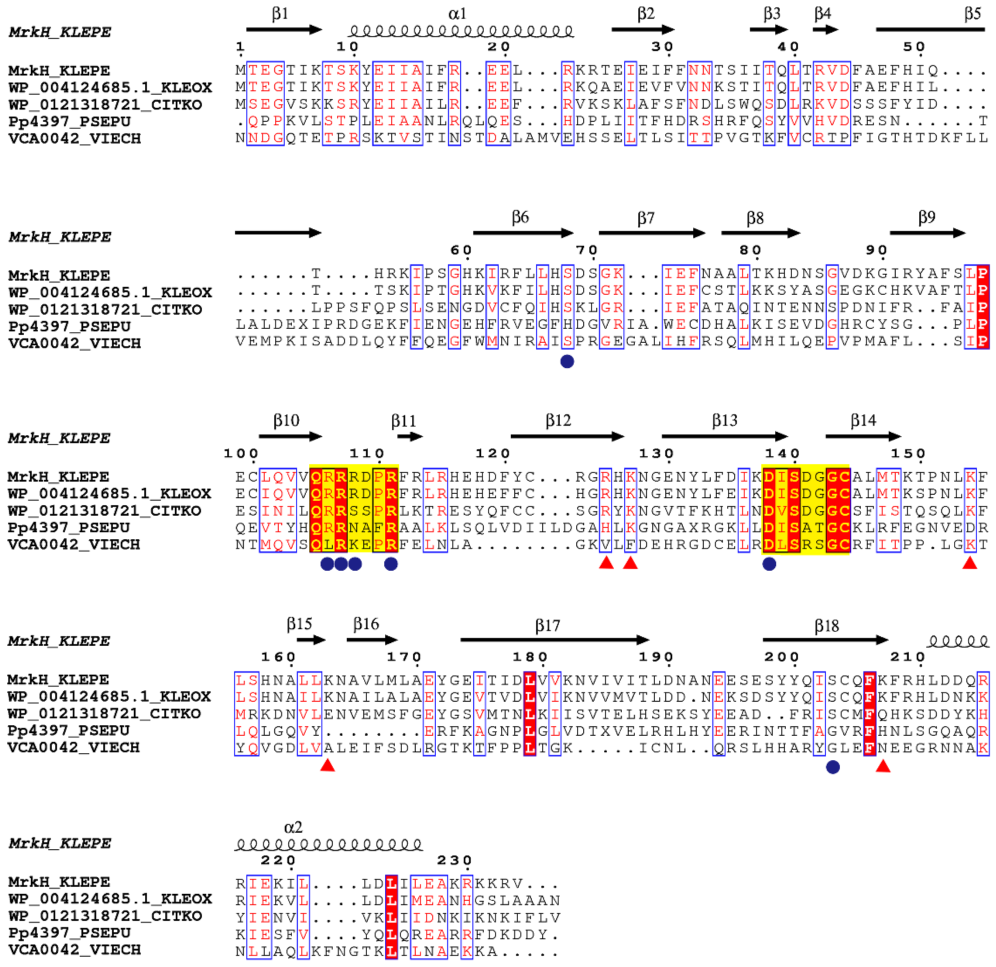

Supplementary Figure S1. Multiple sequence alignment of MrkH and homologues.

The (Q/E)RRXXXR and the DZSXXG moieties are highlighted by orange background and the residues involved in binding c-di-GMP are marked by blue spots. The residues contributing to DNA-binding are marked by red triangles. The multiple sequence alignment was performed using COBALT (www.ncbi.nlm.nih.gov/tools/cobalt) and ESPript 3.0 (http://espript.ibcp.fr/ESPript/cgi-bin/ESPript.cgi). All sequences were downloaded from the NCBI protein-sequence database (http://www.ncbi.nlm.nih.gov). The sequences are (NCBI accession Nos. is given in parentheses) from: Klebsiella pneumoniae (WP_004152886.1), Klebsiella oxytoca (WP_004124685.1), Citrobacter koseri (WP_012131872.1), Pseudomonas Putida KT2440 (WP_010955107.1) and Vibrio cholerae O1 biovar El Tor str. N16961 (WP_001088585.1).

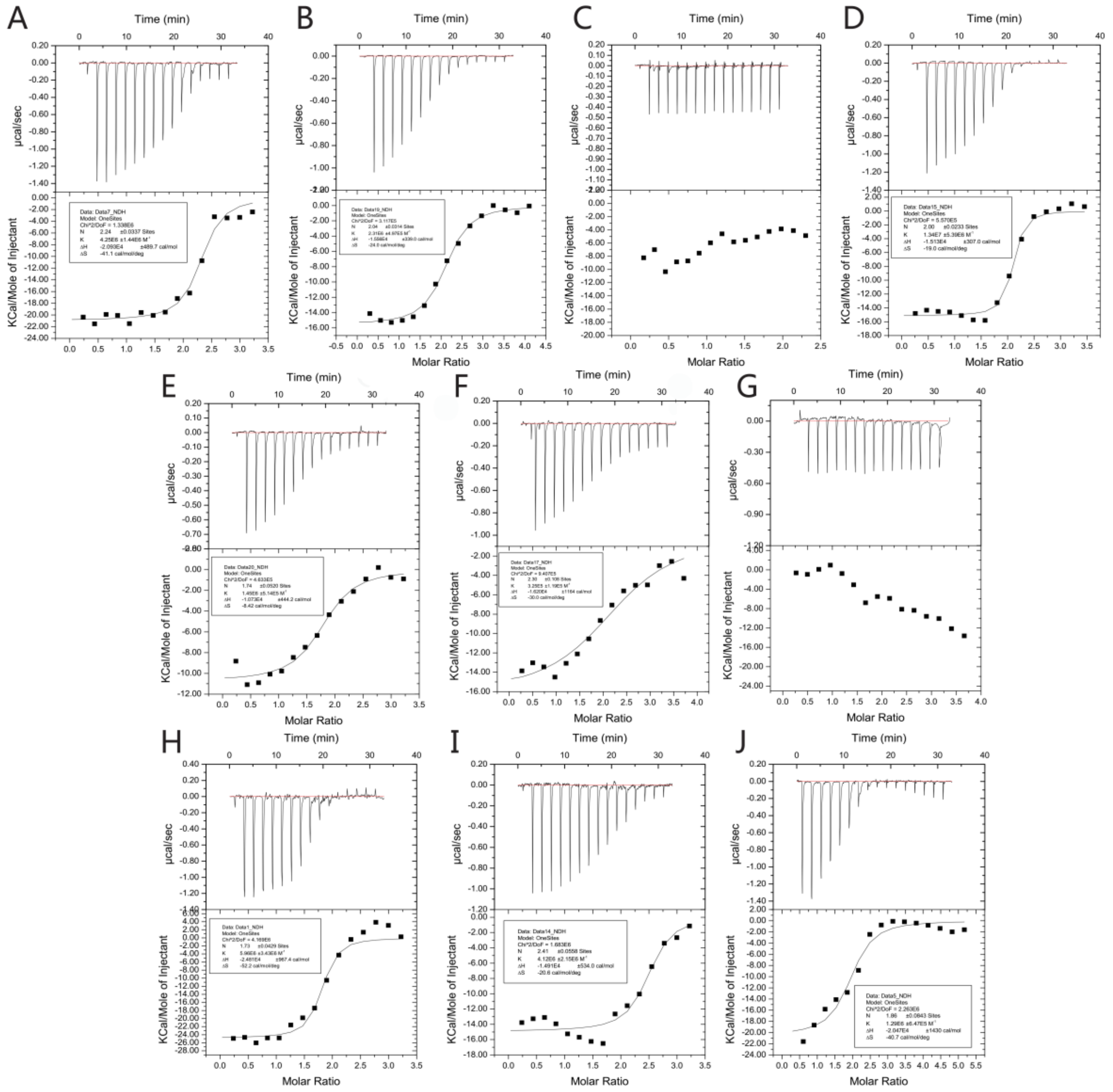

Supplementary Figure S2. Binding of c-di-GMP molecules to MrkH variants.

C-di-GMP solution were titrated into (A) MrkH (B) MrkH105-end (C) MrkH116-end (D) MrkH Q105A (E) MrkH R106A (F) MrkH R107A (G) MrkH R111A (H) MrkH K127A (I) MrkH K154A (J) MrkH K163A.The upper panel shows the raw calorimetric data of the titration, the lower panel shows the corresponding integrated injection heats. The curves represent the best least-squares fits to the one binding site model. Thermodynamic parameters (N, K, ΔG, ΔH) are listed in the small square insets.

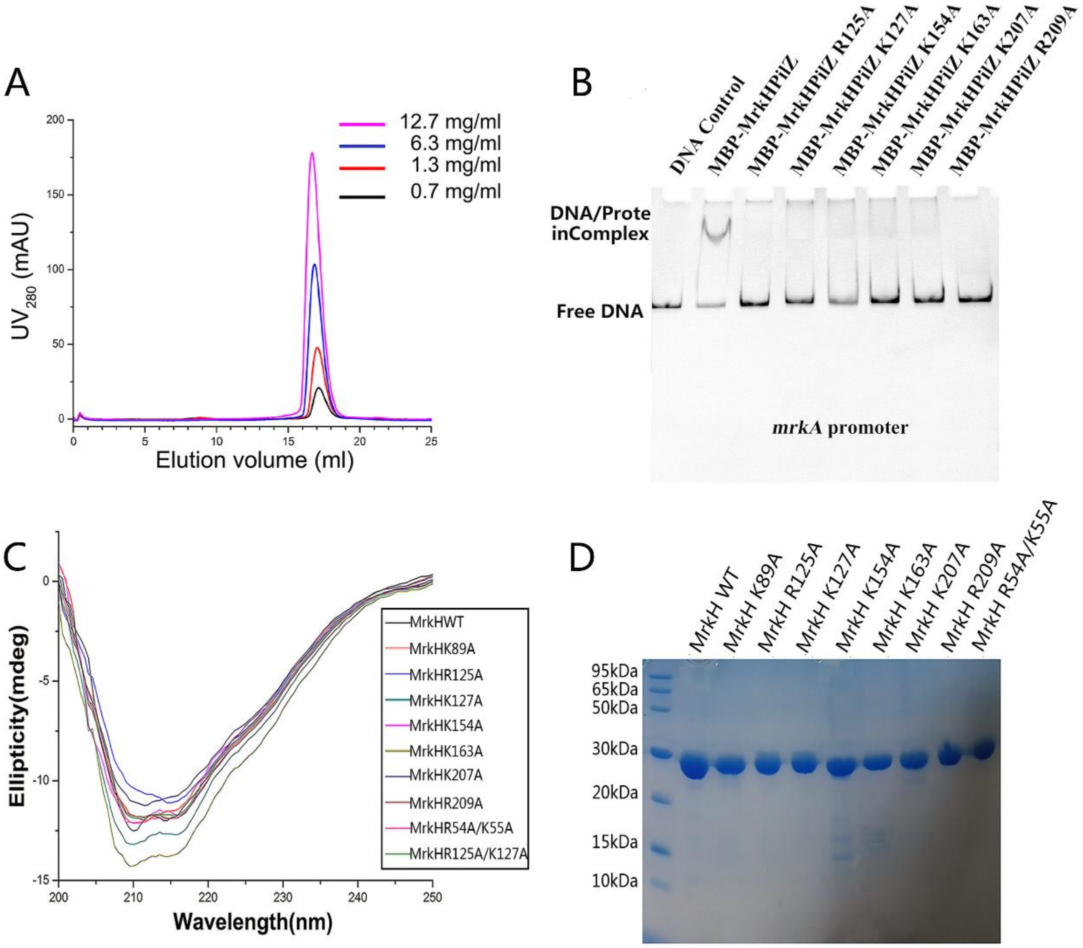

Supplementary Figure S3. Properties of MrkH and MrkH-PilZ variants.

Size-exclusion chromatography of MrkH-PilZ domain at different concentrations. (B) Residues involved in DNA binding are verified by EMSA of MrkH-PilZ and its mutants with the 288bp *mrkA* promoter sequence. (C) CD spectra of MrkH and its mutants. (D) Four µg of MrkH mutants were run in 15% PAA-gels and visualized by coomassie brilliant blue staining.

Supplementary Table 1. ***K_d* values and stoichiometric coefficients for the binding of c-di-GMP to MrkH variants measured by ITC**

	K_d (μM)	Stoichiometric Coefficient（N）
MrkH-WT	0.24 ±0.08	2.24 ±0.03
MrkH-Q105A	0.09 ±0.04	2.00 ±0.02
MrkH-R106A	0.78 ±0.28	1.74 ±0.05
MrkH-R107A	3.55 ±1.30	2.30 ±0.11
MrkH-R111A	NA	NA
$MrkH^{105\text{-}228}$	0.45 ±0.10	2.04 ±0.03
$MrkH^{116\text{-}228}$	NA	NA
MrkH-K127A	0.17 ±0.10	1.73 ±0.04
MrkH-K154A	0.24 ±0.13	2.41 ±0.06
MrkH-K163A	0.77 ±0.38	1.86 ±0.08

Supplementary Table 2. X-ray diffraction data-collection and refinement statistics

Values in parentheses are for the outer resolution shell

	Native MrkH/c-di-GMP complex	I3C-dreivative MrkH/ c-di-GMP complex
Data collection		
Wavelength (Å)	0.9792	1.54
Space group	$P4_12_12$	$P4_12_12$
Unit-cell parameters (Å, °)	a= 68.56, b= 68.56, c= 179.46, α=β=γ=90.0	a= 68.56, b= 68.56, c= 179.46, α=β=γ=90.0
Resolution (Å)	50-2.30(2.38-2.30)	50-3.00（3.11-3.00）
No. of unique reflections	19898	8865
Multiplicity	13.8(14.5)	20.5（19.6）
Completeness (%)	99.9(100)	98.2（97.8）
$\langle I/\sigma(I) \rangle$	31.96(8.45)	72.79（26.06）
R_{merge}† (%)	8.2(41.3)	14.3（48.4）
Refinement		
Resolution range (Å)	37.54-2.30(2.38-2.30)	

R_{work} ‡ / R_{free} § (%)	21.3/26.1
Protein atoms	1930
Ligand atoms	119
Water molecules	88
R.m.s. deviations	
Bonds (Å)	0.009
Angles (°)	0.975
Ramachandran plot (%)	
Most favoured	94.00
Allowed	6.00
Outlier	0.00
Average B factor (Å^2)	
Overall	53.1
Protein atoms	54.5
Ligand atoms	35.3
Solvent atoms	45.6

† $R_{merge} = \sum_{hkl}\sum|I_i(hkl) - <I(hkl)>| / \sum_{hkl}\sum_i I_i(hkl)$, where $I_i(hkl)$ is the intensity of an individual measurement of reflection $I(hkl)$, and $<I(hkl)>$ is the mean intensity of reflection $I(hkl)$. ‡ $R_{work} = \sum_{hkl} \big| |F_{obs}|-|F_{cale}| \big| / \sum_{hkl}|F_{obs}|$, where $F_{obs}$and F_{cale} are the observed and calculated structure factors for reflection *hkl*, respectively. § R_{free} was calculated in the same way as R_{work} but using a randomly selected 5% of the reflections which were omitted from refinement.

Supplementary Table 3. Primers used in EMSA probes of *mrkHI* and *mrkA*.

	**primers**
mrkA-117	5’-GCTGCGCTGTAAACAACCACCCTCGCGT-3’
mrkA+166	5’-AGCACACTGTGAATTCGCATAGAACCAGAAACAT-3’
mrkHI -184	5’-ACTTAAGTTTTTATCCTTCGACCGGTCTCCAG-3’
***mrkHI* +52**	**5’-ATGCATCCCTTGTAAATAGTTGTCGTGAGGCG-3’**

Supplementary Table 4. Oligonucleotide sequences used in FP experiments

**Oligonucleotides**	**Sequences**

P*mrkA*-117to +166	5'-FAM-GCTGCGCTGTAAACAACCACCCTCGCGTTTTCATCTATCAATGGCTGTTTATTAATAGTCGATGGTTATCTGTTATATAACTTAATGAAACGTGAACAAATGTATATTTGTCGGCGAATAAATAGCATTCTTTGACGCCGATAGCACCAGCGAACTTATATTTTTAGGTTCGTTACCTGACGCCTTAAATATTGCGGCTCCCGGTCAGTCGAAGATTCTATTAGCAGTGGCATAAATCAGCATCGCCTGATGTTTCTGGTTCTATGCGAATTCACAGTGTGCT -3'
P*mrkA*-117to -37	5'-FAM-GCTGCGCTGTAAACAACCACCCTCGCGTTTTCATCTATCAATGGCTGTTTATTAATAGTCGATGGTTATCTGTTATATAA -3'
P*mrkA*-117to-37 the 'MrkH box' mutant	5'-FAM-GCTGCGCTGTAAACAACCACCCTCGCGTTTTGAAGTCAATCGTGCTGTTTATTAATAGTCGATGGTTATCTGTTATATAA -3'
80bps random sequence	5'-FAM-ATAAATAGCATTCTTTGACGCCGATAGCACCAGCGAACTTATATTTTTAGGTTCGTTACCTGACGCCTTAAATATTGCGG -3'
**The 'MrkH box'**	**5'-FAM-CATCTATCAATG-3'**

Reference

Adams, P.D., Afonine, P.V., Bunkoczi, G., Chen, V.B., Davis, I.W., Echols, N., Headd, J.J., Hung, L.W., Kapral, G.J., Grosse-Kunstleve, R.W., *et al.* (2010). PHENIX: a comprehensive Python-based system for macromolecular structure solution. Acta Crystallogr D *66*, 213-221.

DeLano, W.L. (2002). The PyMOL molecular graphics system.

Emsley, P., Lohkamp, B., Scott, W.G., and Cowtan, K. (2010). Features and development of Coot. Acta Crystallogr D *66*, 486-501.

Ko, J., Ryu, K.S., Kim, H., Shin, J.S., Lee, J.O., Cheong, C., and Choi, B.S. (2010). Structure of PP4397 Reveals the Molecular Basis for Different c-di-GMP Binding Modes by Pilz Domain Proteins. J Mol Biol *398*, 97-110.

Laskowski, R.A., MacArthur, M.W., Moss, D.S., and Thornton, J.M. (1993). PROCHECK: a program to check the stereochemical quality of protein structures. J Appl Crystallogr *26*, 283-291.

Leslie, A.G.W., Powell, H.R., Winter, G., Svensson, O., Spruce, D., McSweeney, S., Love, D., Kinder, S., Duke, E., and Nave, C. (2002). Automation of the collection and processing of X-ray diffraction data - a generic approach. Acta Crystallogr D *58*, 1924-1928.

Murshudov, G.N., Skubak, P., Lebedev, A.A., Pannu, N.S., Steiner, R.A., Nicholls, R.A., Winn, M.D., Long, F., and Vagin, A.A. (2011). REFMAC5 for the refinement of macromolecular crystal structures. Acta Crystallogr D *67*, 355-367.

Whitney, J.C., Whitfield, G.B., Marmont, L.S., Yip, P., Neculai, A.M., Lobsanov, Y.D., Robinson, H., Ohman, D.E., and Howell, P.L. (2015). Dimeric c-di-GMP is Required for Post-translational Regulation of Alginate Production in Pseudomonas aeruginosa. J Biol Chem *290*, 12451-12462.

Xia, Y., Chu, W., Qi, Q., and Xun, L. (2015). New insights into the QuikChangeTM process guide the use of Phusion DNA polymerase for site-directed mutagenesis. Nucleic Acids Res *43*, e12-e12.
